# Glycemic Characteristics and Clinical Outcomes in Patients with COVID-19 Admitted to Referral Shahid Sayad Shirazi Hospital in Gorgan, North of Iran

**DOI:** 10.1155/2023/1374819

**Published:** 2023-11-06

**Authors:** Saba Kordrostami, Maryam Zahedi, Abdolhalim Rajabi, Shahab Eskandari-Nejad

**Affiliations:** ^1^Clinical Research Development Unit (CRDU), 5 Azar Hospital, Golestan University of Medical Sciences, Gorgan, Iran; ^2^Department of Internal Medicine, Endocrinology and Metabolic Disorders, Clinical Research Development Unit (CRDU), Sayyad Shirazi Hospital, Golestan University of Medical Sciences, Gorgan, Iran; ^3^Department of Biostatistics and Epidemiology, School of Health, Golestan University of Medical Sciences, Gorgan, Iran; ^4^Department of Internal Medicine, School of Medicine, Golestan University of Medical Sciences, Gorgan, Iran

## Abstract

**Background:**

Diabetes mellitus (DM) is one of the most common chronic diseases, the main manifestation of which is hyperglycemia, and is accompanied by many complications. Since the outbreak of the coronavirus disease 2019 (COVID-19) pandemic, several studies have reported the occurrence of various complications associated with different degrees of hyperglycemia in COVID-19 patients. The aim of the present study was to investigate the glycemic characteristics and clinical outcomes in patients with COVID-19.

**Methods:**

In this cross-sectional study, 418 patients with COVID-19 were evaluated in terms of hyperglycemia and its related factors, as well as the relationship between hyperglycemia and the outcome of the disease. Data were statistically analyzed using SPSS software.

**Results:**

In the present study, 350 (83.7%) out of 418 hospitalized patients with COVID-19 had hyperglycemia and 193 (55.1%) of the patients with hyperglycemia were women. 169 (48.4%) of patients with hyperglycemia during hospitalization were already diabetic. The mean age was higher in COVID-19 patients with hyperglycemia (*P* < 0.001), and systolic blood pressure and respiratory rate were also higher in them (*P* = 0.005 and *P* = 0.013, respectively). In patients with hyperglycemia, oxygen saturation (SpO_2_) at the time of admission and discharge was lower than other patients (*P* < 0.001). The frequency of hypertension in the patients with hyperglycemia was significantly higher than in nonhyperglycemic patients (*P* < 0.001 vs. 0.014). The estimated glomerular filtration rate (eGFR) of hyperglycemic patients was significantly lower than other patients (*P* < 0.001). Also, there was a significant inverse relationship between eGFR values and fasting (FBS) and random blood sugar (BS) (*r* = 0.328 and *r* = 0.310, *P* < 0.001). On the other hand, there was a direct relationship between FBS and random BS in patients with hyperglycemia with the dose of corticosteroids (*r* = 0.146 and *r* = 0.158, *P* < 0.01). In total, 8.2% of the patients died, although the FBS and random BS and a history of DM were not risk factors for the death of patients (*P* < 0.05).

**Conclusion:**

The findings of our study showed that hyperglycemia is highly prevalent in hospitalized patients with COVID-19. Hyperglycemia in previously nondiabetics appears to be associated with decreased eGFR in COVID-19 patients.

## 1. Introduction

Diabetes mellitus (DM) includes a group of common metabolic disorders characterized by the presence of hyperglycemia. Currently, 425 million people in the world and 8 million people in Iran are diabetic, and the annual incidence of DM is 4 million people [1, 2]. DM is associated with multiple acute and chronic complications and is one of the most important causes of mortality and morbidity worldwide. Vascular complications as a result of damage to both the macrovascular (cardiovascular and cerebrovascular diseases (CVDs)) and microvascular (diabetic kidney injury (DKI), diabetic retinopathy (DR), and diabetic neuropathy (DN)) systems are the most important causes of mortality and morbidity in diabetic patients. These complications, in addition to many social problems, impose a large financial burden on healthcare systems in developed and developing countries [3, 4].

Coronavirus disease 2019 (COVID-19) is a pandemic infection that has affected more than 550 million people worldwide and caused more than 6 million deaths across the world. COVID-19 by causing severe acute respiratory syndrome (SARS) is recognized as a global public health problem. Among the people who are hospitalized because of COVID-19, more than a quarter need to be admitted to the intensive care unit (ICU), which constitutes about 5 to 10 percent of all infected cases. The mortality rate for severe cases of the disease has been reported to vary from 7 to 62% [5, 6].

The exacerbation of hyperglycemia in patients with preexisting DM or the new onset of hyperglycemia in previously nondiabetics is one of the most important disorders that can worsen the prognosis of COVID-19 as DM and hyperglycemia were also recognized as risk factors for poor prognosis during previous SARS-CoV-2 epidemic [7, 8]. Reports of patients with SARS-CoV-2 infection showed that 45–52% presented with hyperglycemia, while at most 10% of them had a previous history of DM. It is important to highlight that hyperglycemia and COVID-19 may have a bidirectional relationship [8, 9]. On the one hand, DM is characterized by a chronic, proinflammatory state, so diabetic patients are more likely to develop a catastrophic inflammatory response to COVID-19 infection. Also, patients with DM often present multiple comorbidities, such as CVD, obesity, and metabolic syndrome, which may mediate the increased risk for complicated COVID-19 [10, 11].

Given that a better description of the glycemic profile of patients with COVID-19 infection in critical patients can be useful in guiding clinical decisions, since hyperglycemia may lead to more infectious complications. The aim of the present study was to investigate the glycemic characteristics and clinical outcomes in patients with COVID-19 admitted to Shahid Sayad Shirazi Hospital, a referral center in Gorgan city, north of Iran.

## 2. Materials and Methods

### 2.1. Study Design

This cross-sectional study was conducted on patients with a definite diagnosis of COVID-19 infection who were admitted due to the presence of at least one case of severe respiratory symptoms, acute complications, or underlying diseases. Exclusion criteria included previous history of advanced heart, respiratory, renal, and hepatic failure as well as missing data required for review. Finally, 418 patients with COVID-19 infection hospitalized from March 2021 to February 2022 in the referral to Shahid Sayad Shirazi Hospital in Gorgan city, north of Iran were evaluated. In all these 418 participants, the severity of the disease, the type, duration, and dose of total corticosteroids; the type of anti-COVID-19 treatment, the occurrence of acute complications, and the final outcome of hospitalization (either recovery or death); and in people with a history of DM or recent hyperglycemia, the average daily dose of insulin was investigated.

### 2.2. Definitions

The definitive diagnosis of COVID-19 infection was determined by the diagnosis of the disease by an infectious disease specialist or a lung subspecialist according to a positive polymerase chain reaction (PCR) or high-resolution computed tomography (HRCT) chest imaging findings. Based on the American Diabetes Association- (ADA-) 2021 criteria history of DM with fasting blood sugar (FBS) ≥126 mg/dL and/or random blood sugar (BS) ≥ 200 mg/dL and/or hemoglobin A1c (HbA1c) ≥6.5% and also, history of pre-DM with FBS: 100–125 mg/dL and/or random BS: 140–199 mg/dL and/or HbA1c: 5.6–6.4% in previous tests, the diagnosis was considered in advance with the opinion of an internist or endocrinologist, while recent hyperglycemia in a person with no previous history of DM was considered according to FBS ≥126 mg/dL and recent pre-DM with FBS: 100–125 mg/dL following hospitalization. The severity of the disease was considered according to the occurrence of at least one of the following: the need for an intensive care unit (ICU), the need for a ventilator, a decrease in the level of consciousness, and a decrease in oxygen saturation (SpO_2_) < 90%. The incidence of acute complications was considered with adverse disease outcomes, including acute respiratory, heart, liver failure, deep vein thrombosis (DVT), pulmonary thromboembolism (PTE), and mucormycosis.

### 2.3. Data Collection

For all COVID-19 patients who were included in the study, demographic data including age, gender, and body mass index (BMI) were collected from an electronic medical record review. In the present study, the average FBS and random BS of the patients were calculated as the average of FBS and BSs of the first 5 days of hospitalization. In patients who were hospitalized for less than 5 days, their FBS and random BS averages were obtained based on the average number of measured days.

In all the studied hospitalized patients, the severity of the disease, the type, duration, and dose of total corticosteroid; the type of anti-COVID-19 treatment, the occurrence of acute complications, the final result of hospitalization (recovery or death), and history of hypertension (HTN); and in people with a history of DM or recent hyperglycemia, the average daily dose of insulin was recorded. Furthermore, symptoms at presentation, all comorbid conditions, medications administered during the hospitalization, and laboratory parameters (serum creatinine and BS level) at admission were also abstracted. The estimated glomerular filtration rate (eGFR) was calculated by the modification of diet in renal disease (MDRD) equation (eGFR (mL/min/1.73 m^2^) = 175 × (sCr)-1.154 × (age)-0.203 × (0.742 if female) × (1.212 if African American)) [12]. In this study, we evaluated the treatment variables including ICU admission, ventilator, and oxygen therapy.

### 2.4. Statistical Analysis

Statistical analysis was performed with SPSS version 19, with *P* values of less than 0.05 as a threshold of significance. Frequencies and percentages were reported for categorical variables and mean and standard deviations for numerical continuous variables. The chi-square test was used to compare qualitative variables. For quantitative variables, first, their normality was checked using the Kolmogorov–Smirnov test. The one-way ANOVA test (Kruskal–Wallis in nonparametric condition) test was used in three or more groups. Friedman's test was used to compare glycemic changes over time. Correlation between quantitative parameters was performed using Spearman's correlation coefficient test.

## 3. Results

### 3.1. Patient Characteristics

This study consisted of 418 patients diagnosed with COVID-19 where 190 (45.5%) were male and 228 (54.5%) were female. The average age and BMI level of the studied patients were 51.61 ± 14.97 years (with a range of 14–90) and 28.46 ± 5.62 kg/m^2^ (range of 17–58). The clinical indicators and outcomes of COVID-19 patients are shown in 1. As can be seen in Table 1, the most important symptoms of patients admitted to the hospital were shortness of breath and cough. 5.5% of patients had a history of an important disease including heart, kidney, or lung failure. 22% of patients showed some degree of pulmonary involvement in HRCT chest imaging. The severity of COVID-19 was seen in 9.8% of patients. 399 (95.5%) of the patients received dexamethasone. The predominant anti-COVID-19 treatment in 395 (94.5%) patients was remdesivir. 30 (7.2%) of the patients in our study died. Furthermore, the glycemic profile of hospitalized COVID-19 patients is shown in Table 2. Out of 418 hospitalized COVID-19 patients, 350 (83.7%) of them had hyperglycemia, 32 (7.7%) had increased BS to the level of pre-DM, and 36 (8.6%) had normal BS, while only 168 (40.2%) people were already diabetic.

### 3.2. Demographic and Clinical Characteristics of COVID-19 Patients Based on Glycemia

The results indicated that the average age of patients with hyperglycemia was significantly higher than patients with pre-DM and normoglycemia (*P* = 0.034 and *P* < 0.001, respectively), but there was no significant difference between pre-DM and normoglycemic patients. Systolic blood pressure (SBP) of hyperglycemic patients was significantly higher than that of normoglycemic patients (*P* = 0.007), and their respiratory rate (RR) was significantly higher than that of pre-DM patients (*P* = 0.039). It should be mentioned that SpO_2_ at the time of admission of hyperglycemic patients was significantly lower than both groups of patients with pre-DM and normoglycemia (*P* < 0.001), and SpO_2_ at the time of discharge was significantly lower than that of normoglycemic patients (*P* = 0.001) and there was no significant difference in pre-DM patients. Furthermore, the mean serum creatinine (sCr) of hyperglycemic patients was higher than that of normoglycemic patients (*P* = 0.013), but it was not different from pre-DM patients. However, the eGFR of hyperglycemic patients was significantly lower than both normoglycemic (*P* < 0.001) and pre-DM (*P* = 0.014) patients (Table 3).

**Figure 1:**
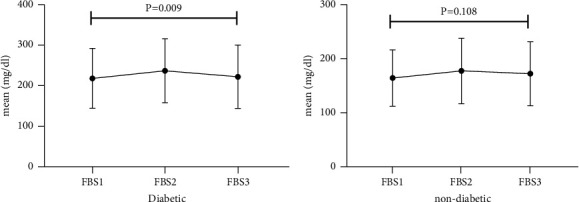
Trend of hyperglycemia exacerbation in patients with COVID-19 according to previous history of DM.

The trend of hyperglycemia exacerbation in patients with COVID-19 according to the previous history of DM was performed by Friedman's two-way analysis of variance by ranks' test (Figure 1). The results of our study showed that in patients with a history of DM after hospitalization due to COVID-19, during the treatment process, FBS first increased and then decreased to its baseline level, which was statistically significant (*P* = 0.009).

### 3.3. Subset Analysis

The results of the present study showed that there is a positive and significant correlation between the dose of corticosteroid prescribed and the increase in FBS and random BS in patients through the Spearman correlation coefficient test (*r* = 0.146; *P* = 0.004, *r* = 0.156; *P* = 0.002, respectively) (Table 4).

In addition, in our study, it was found that the relationship between the mean values of FBS and sCr in the normoglycemic patients was direct, however, no relationship between FBS and eGFR was observed. In the pre-DM patients, the relationship between FBS and random BS with sCr was direct and with eGFR was inverse and significant. Also, in hyperglycemic patients, the relationship between the mean values of FBS and random BS with sCr was direct and with eGFR was inverse and significant (Table 5).

**Figure 2:**
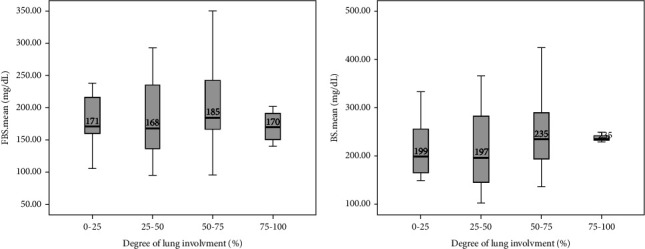
Comparison of mean FBS (a) and random BS (b) based on the severity of lung involvement in COVID-19 patients.

**Figure 3:**
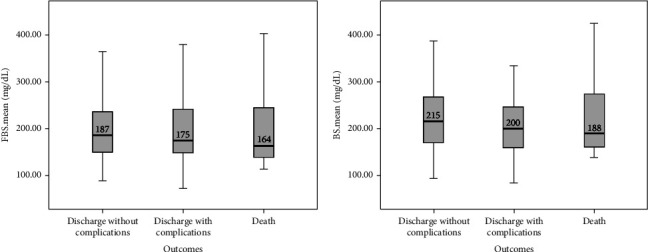
Comparison of mean FBS (a) and random BS (b) based on the outcome of the disease in COVID-19 patients.

In this study, the comparison between FBS and random BS of patients with COVID-19 based on the severity of lung involvement was made by the Kruskal–Wallis test and the results did not show any significant difference (Figure 2). In addition, the comparison between mean FBS and random BS of patients with COVID-19 based on the outcome of the disease was made by the Kruskal–Wallis test and the results did not show any significant difference (Figure 3). Finally, logistic regression analysis did not show mean FBS, mean random BS, history of DM, and hyperglycemia as risk factors for the death of COVID-19 patients (*P* < 0.05).

## 4. Discussion

SARS-CoV-2, as the causative agent of the COVID-19 disease, became a public health emergency of international concern soon after the start of the outbreak in Wuhan, China. DM has emerged as an important risk factor for more severe COVID-19 illness and death [13, 14]. Our study aimed to investigate the glycemic characteristics and clinical outcomes in patients with COVID-19. In the current study, with a random survey of 418 hospitalized patients with a confirmed diagnosis of COVID-19, a total of 168 (40.2%) patients mentioned a history of type 2 DM and were still being treated. Regardless of the previous history of DM, 350 (83.7%) of the patients were in the hyperglycemic group and 32 (7.7%) were in the pre-DM group.

The findings of our study showed that the age of hyperglycemic patients was significantly higher than pre-DM and normoglycemic patients, but there was no significant difference between normoglycemic and pre-DM patients. The SBP of hyperglycemic patients was statistically higher than that of normoglycemic patients, but it was clinically within the normal range. The RR of hyperglycemic patients was significantly higher than that of pre-DM patients. SpO_2_ at the time of admission of patients with hyperglycemia was significantly lower than both normoglycemic and pre-DM patients, while SpO_2_ at the time of discharge of hyperglycemic patients was lower than that of normoglycemic patients. The eGFR of hyperglycemic patients was significantly lower than both normoglycemic and pre-DM patients. Note that, in patients with hyperglycemia and pre-DM there was a significant direct relationship between the averages of FBS and random BS with sCr, and their relationship with eGFR was inversely significant. In patients with a history of DM after hospitalization due to COVID-19, FBS first increased and then decreased during the treatment process, but this trend was not observed in patients with hyperglycemia without a history of DM. Finally, the outcome of death was observed in 8.2% of COVID-19 patients, although the increase in BS was not a risk factor for the death of these patients.

A study by Bode et al. [13] showed that COVID-19 patients with hyperglycemia had higher mortality and length of hospital stay than patients without hyperglycemia. They showed that the prevalence of hyperglycemia in patients with COVID-19 was 40.2% and the eGFR (63.7 ml/min) of hyperglycemic patients was significantly lower than nonhyperglycemic subjects. In our study, 83.4% of patients had hyperglycemia (46% with a previous history of DM), which was more than twice the amount observed in Bode et al.'s study. In our study, like the study of Bode et al., the eGFR (63.6 ml/min) of hyperglycemic patients was significantly lower than nonhyperglycemic subjects.

In Sardu et al.'s study [15], it was shown that 42.2% of COVID-19 patients had hyperglycemia, and patients with hyperglycemia were at a higher risk of severe disease than nonhyperglycemic patients. In the present study, the prevalence of hyperglycemia was 83.7% and no correlation between hyperglycemia and disease severity was observed, which is inconsistent with the findings of the study by Sardu et al. Another study showed that hyperglycemia on the first day of hospitalization was the best predictor of radiographic imaging of SARS-CoV-2, regardless of the past medical history of DM [16]. However, in our study, no difference was observed between FBS and random BS of patients based on the severity of lung involvement, which is inconsistent with the findings of this study. One of the reasons for this discrepancy can be related to the lack of completion of the patients' files in terms of radiographic imaging findings in our study.

In the study of Fadini et al. [17], 25.6% of patients with COVID-19 had hyperglycemia, of which 80% had DM before contracting COVID-19. Higher glucose levels on admission were associated with the severity of COVID-19, with a stronger association among patients without a history of DM compared to those with preexisting DM. In the present study, it was shown that there is no relationship between FBS and random BS of patients with their morbidity at the time of discharge.

In their study, Liu et al. [18] stated that poor BS control in diabetic patients with COVID-19 is associated with adverse outcomes for them. In our study, it was also shown that the increase in BS in COVID-19 patients without a history of DM is related to a significant decrease in eGFR in them, while such a relationship was not observed in diabetic patients. The reason for observing such a phenomenon can be described as that diabetic patients prevent adverse microvascular effects caused by increased BS on the kidney due to the regular use of antidiabetic drugs, while a high percentage of nondiabetic patients with COVID-19 who suffer from newly diagnosed hyperglycemia and do not receive antidiabetic drugs. In fact, some people with DM or pre-DM are unknown and abandoned. Another study showed that in diabetic patients with COVID-19, hypertension, coronary artery disease, and chronic kidney disease were significantly more common than in COVID-19 patients without type 2 DM [19]. The findings of our study also showed that chronic HTN and decreased glomerular function were significantly associated with hyperglycemia. Finally, in a study by Douin et al. [20], it was shown that the administration of corticosteroids to hospitalized COVID-19 patients under ventilation was associated with hyperglycemia. The findings of our study also showed that there is a direct and positive correlation between the dose of corticosteroid prescribed and the BS of the COVID-19 patients.

## 5. Conclusion

The findings of our study showed that acute hyperglycemia and DM are highly prevalent in patients with COVID-19. Older age and SBP, a history of HTN, decreased SpO_2_ during hospitalization and discharge, as well as eGFR were associated with hyperglycemia in COVID-19 patients. In the present study, there was no correlation between the death of COVID-19 patients and hyperglycemia. Finally, undetected hyperglycemia appears to be associated with decreased glomerular function in patients with COVID-19.

## Figures and Tables

**Table 1:** The clinical profile and outcomes in COVID-19 patients.

Factors	Frequency (%)
*Symptoms*	
Shortness of breath	88 (21.2)
Cough	121 (29.1)
Diarrhea and vomiting	21 (5)
Weakness and lethargy	44 (10.6)
Fever	35 (8.4)
Shortness of breath and cough	107 (25.7)
*Previous disease history*	
Heart failure	16 (3.8)
Respiratory failure	1 (0.2)
Kidney failure	5 (1.2)
*Pulmonary involvement*	
<25%	17 (4.1)
25–50%	39 (9.3)
50–75%	31 (7.4)
≥75%	5 (1.2)
*Severe disease*	
ICU admission	14 (3.3)
Need a ventilator	15 (3.6)
Loss of consciousness	5 (1.2)
SpO_2_ drops to less than 90%	7 (1.7)
*Corticosteroid administration*	
Dexamethasone	399 (95.5)
Methylprednisolone	17 (4.1)
*Anti-COVID-19*	
Remdesivir	395 (94.5)
Actemra	18 (4.3)
Other	3 (0.7)
*Outcomes*	
Discharge without complications	338 (80.8)
Discharge with complications	50 (12)
Death	30 (7.2)

Quantitative variables	Mean ± SD (range)

SBP(mmHg)	121.6 ± 16.4 (43–195)
DBP (mmHg)	75.1 ± 11.6 (10–185)
PR (/minute)	90.5 ± 16.1 (49–200)
RR (/minute)	19 ± 3.8 (10–40)
Temperature (C°)	37.3 ± 1.5 (18–40)
SpO_2_ at time of admission (%)	92 ± 6.3 (37–100)
SpO_2_ at discharge (%)	93.5 ± 7.5 (40–100)
Serum creatinine (mg/dl)	1.22 ± 0.42 (0.4–3.8)
eGFR (mL/min/1.73 m^2^)	64.8 ± 20.3 (14.4–136.3)

BMI, body mass index; SBP, systolic blood pressure; DBP, diastolic blood pressure; PR, pulse rate; RR, respiratory rate; SpO_2_, blood oxygen saturation level; eGFR, estimated glomerular filtration rate.

**Table 2:** The glycemic profile of COVID-19 patients.

Factors	Frequency (%)
History of DM	168 (40.2)
Glycemic category based on FBS (mg/dl) during hospitalization regardless of the previous history of DM	
Normal (FBS < 100)	36 (8.6)
Pre-DM (100 < FBS < 125)	32 (7.7)
DM (FBS < 125)	350 (83.7)
*Drugs used for DM*	
Oral except metformin	17 (10.2)
Metformin	70 (42.2)
Insulin	50 (30.1)
Metformin + oral	23 (13.9)
Insulin + oral	6 (3.6)

Factors	Mean ± SD (range)

FBS during hospitalization (mg/dl)	195.2 ± 63.6 (73–481)
Random BS during hospitalization (mg/dl)	219.9 ± 68.2 (83–500)
Average insulin dose during hospitalization (unit)	9.1 ± 5.9 (2–40)

DM, diabetes mellitus; FBS, fasting blood sugar; BS, blood sugar.

**Table 3:** Comparison of demographic and clinical characteristics of COVID-19 patients based on glycemic classification.

Qualitative factors^+^	Hyperglycemia (%) *N* = 350	Pre-DM (%) *N* = 32	Normoglycemia (%) *N* = 36	*P* value^+^
*Gender* ^*+*^				
Male	157 (82.6)	15 (7.9)	18 (9.5)	0.828
Female	193 (84.6)	17 (7.5)	18 (7.9)	
History of hypertension^+^	130 (39)	8 (25)	6 (16.7)	0.012
*Anti-COVID-19* ^*+*^				
Remdesivir	331 (79.57)	30 (7.21)	34 (8.17)	0.045
Actemra	16 (3.85)	2(0.48)	0 (0)	
Other	1 (0.24)	0 (0)	2 (0.48)	

Quantitative factors^++^	Hyperglycemia (mean ± SD)	Pre-DM (mean ± SD)	No diabetes (mean ± SD)	*P* value^+^

Age (years)^+++^	52.3 ± 13.9	45.2 ± 8.3	41.4 ± 16.3	0.001
BMI (kg/m^2^)^++^	27.4 ± 6	25.5 ± 6	27.1 ± 6	0.225
SBP (mmHg)^++^	120 ± 20	120 ± 21	110 ± 30	0.007
DBP (mmHg)^++^	75 ± 10	70 ± 12.5	70 ± 23.75	0.395
PR (/minute)^++^	90 ± 20	84 ± 14	84 ± 17.25	0.154
RR (/minute)^++^	18 ± 2	18 ± 3	18.5 ± 1.75	0.039
Temperature (°C)^++^	37.1 ± 0.68	37 ± 1.2	37.25 ± 1.38	0.517
SpO2 at time of admission (%)^++^	93 ± 6	94 ± 6	96 ± 3	0.001
SpO_2_ at discharge (%)^++^	95 ± 4	96 ± 3	96.5 ± 2.75	0.001
sCr (mg/dl)^++^	1.10 ± 3.4	1.10 ± 0.2	1.05 ± 0.3	0.013
eGFR (mL/min/1.73 m^2^)^++^	62.51 ± 22	72.88 ± 27.1	73.65 ± 27.6	0.001

BMI, body mass index; SBP, systolic blood pressure; DBP, diastolic blood pressure; PR, pulse rate; RR, respiratory rate; SpO_2_, blood oxygen saturation level; sCr, serum creatinine; eGFR, estimated glomerular filtration rate; ^+^chi-square test was used; ^++^Kruskal–Wallis test was used; ^+++^ANOVA test was used.

**Table 4:** The relationship between patients' blood sugar and corticosteroid medication in COVID-19 patients.

Corticosteroid	Mean FBS levels		Mean BS levels	
	*P* value^+^	*r* ^+^	*P* value^+^	*r* ^+^
Drug dose (mg)	0.004	0.146	0.002	0.158
Duration of administration (day)	0.431	−0.04	0.130	−0.076
Dose × duration (mg × day)	0.335	0.049	0.699	0.019

FBS, fasting blood sugar; BS, random blood sugar; ^+^Spearman correlation coefficient test was used. Dose × duration, the number obtained by multiplying the dose of corticosteroid used (equivalent to dexamethasone (mg)) by the duration of use (days).

**Table 5:** The relationship between glycemic status during hospitalization and renal function in COVID-19 patients regardless of the previous history of DM.

Glycemic classification			sCr	eGFR
Hyperglycemia	Mean FBS levels	*P* value^+^	0.001	0.001
		*r* ^+^	0.250	−0.328
	Mean BS levels	*P* value^+^	0.001	0.001
		*r* ^+^	0.204	−0.310

Pre-DM	Mean FBS levels	*P* value^+^	0.031	0.020
		*r* ^+^	0.382	−0.411
	Mean BS levels	*P* value^+^	0.038	0.018
		*r* ^+^	0.388	−0.436

Normoglycemia	Mean FBS levels	*P* value^+^	0.010	0.462
		*r* ^+^	0.738	−0.248
	Mean BS levels	*P* value^+^	0.168	0.846
		*r* ^+^	−0.279	0.040

sCr, serum creatinine; eGFR, estimated glomerular filtration rate; Pre-DM, prediabetes mellitus; FBS, fasting blood sugar; BS, blood sugar; ^+^Spearman correlation coefficient test was used.

## Data Availability

The data used to support the findings of the study are openly available in one of the repositories or will be available on request from the corresponding author by this journal representative during submission or after publication.
